# Modulation of Mucin Secretion in the Gut of Young Pigs by Dietary Threonine and Non-Essential Amino Acid Levels

**DOI:** 10.3390/ani12030270

**Published:** 2022-01-22

**Authors:** Ewa Święch, Anna Tuśnio, Marcin Taciak, Marcin Barszcz

**Affiliations:** Department of Animal Nutrition, The Kielanowski Institute of Animal Physiology and Nutrition, Polish Academy of Sciences, Instytucka 3, 05-110 Jabłonna, Poland; a.tusnio@ifzz.pl (A.T.); m.taciak@ifzz.pl (M.T.); m.barszcz@ifzz.pl (M.B.)

**Keywords:** mucins, mucosa, digesta, sugars, gut, pigs, threonine, wheat gluten

## Abstract

**Simple Summary:**

The mucus layer is an important part of the system protecting the gut against injuries and bacterial infections. The main components of mucus responsible for its properties are mucins. They are large glycoproteins with a protein core rich in threonine (Thr) and many sugar side chains that differ in structure and affect mucin functions. Diet composition affects the amount of secreted mucins and their quality. Therefore, the aim of the study was to determine the effect of Thr and wheat gluten (WG) protein, added as a source of non-essential amino acids, on the content of tissue and luminal mucins in different parts of the intestine of young pigs. Results showed that tissue and luminal mucin content was only affected by WG levels in the duodenum and middle jejunum, and in the proximal colon, respectively. The effect of WG on luminal mucin content in the proximal colon depended on the analytical method applied.

**Abstract:**

The aim of the study was to determine the effect of threonine (Thr) and non-essential amino acid (NEAA) levels on mucin secretion and sugar composition of digesta and crude mucin preparations analyzed in different segments of the gut in young pigs. A two-factorial experiment was conducted on 72 pigs using the following factors: Thr level (5.1, 5.7, 6.3 and 6.9 g standardized ileal digestible(SID) Thr/kg) and wheat gluten (WG) level used as a source of NEAA (20.4, 40.4 and 60.4 g WG protein in WG20, WG40 and WG60 diets, respectively). Mucin content was affected only by WG level. Tissue mucin content in the duodenum was higher in WG60 pigs than in WG20 and WG40 pigs, whereas in the middle jejunum was higher in WG40 and WG60 pigs than in WG20 pigs. In contrast, luminal crude mucin content in the proximal colon was lower in WG60 pigs compared to WG40 pigs. The lowest and highest Thr levels reduced arabinose and xylose contents and increased glucose content in ileal digesta. The highest WG level reduced arabinose and xylose contents and increased glucose content in ileal digesta. The lowest WG level increased mannose content in ileal digesta. WG60 level decreased the content of arabinose and galactose compared to lower WG levels in colonic digesta. Arabinose content was higher, while glucose and galactose contents were lower in crude mucin preparations isolated from colonic digesta in pigs fed diets containing the highest Thr level. The content of tissue mucin was higher in the ileum and proximal colon and lower in the duodenum than in the middle jejunum, whereas luminal mucin content was lower in the proximal colon than in the ileum. Ileal digesta contained less arabinose and glucose and more galactose as compared to colonic digesta. In conclusion, no effect of dietary Thr levels on mucin secretion in the gut of young pigs was found. Wheat gluten added to the diet with adequate Thr content positively affected mucin secretion only in the duodenum and middle jejunum.

## 1. Introduction

The gastrointestinal epithelium is covered with a viscoelastic layer of mucus, which lubricates and protects mucosa against bacterial, chemical and mechanical injuries [1]. Mucins are the main component of the mucus layer responsible for its protective functions [2]. Mucins are large glycoproteins with a molecular weight ranging from 1 to 40 MDa, which enables viscous gel formation [3]. Mucins contain a protein backbone and a large number of oligosaccharide side chains. Five different sugars are found in mucins: *N*-acetylgalactosamine, *N*-acetylglucosamine, galactose, fucose and sialic acids. Depending on sugar type in chains, mucins can be divided into acidic and neutral types. Acidic mucins play a special protective function because they increase mucus resistance against bacterial enzymes [4]. Mucins from different segments of gastrointestinal tract vary in amino acid (AA) and sugar composition.

The optimal protective functions of the mucus layer depend on both quantitative (layer thickness) and qualitative (sugar composition of carbohydrate chains) properties of mucins. The mucus gel is in a dynamic balance between mucin synthesis and secretion from goblet cells of the underlying epithelium and erosion on the luminal side releasing mucins into the gut lumen [5].

Mucin can be a significant source of endogenous losses of protein and AA, such as Thr, proline, glycine and serine. Large amounts of dietary Thr (up to 50%) are retained by the intestine for mucin secretion [6,7]. It has been shown that not only insufficiency, but also a surplus of dietary Thr decreased the fractional synthesis rate of intestinal mucins [8]; it also down-regulated the expression of mucin genes in the small intestine, leading to disorders of the intestinal mucosal barrier (including villus atrophy, increased apoptosis and reduced number of goblet cells) [9]. Dietary Thr imbalance can adversely modify the architecture of villi, crypts and mucin types in the small intestine of young pigs.

Non-essential AA (NEAA) may also play a key role in maintaining intestinal structure and function. Most of the dietary glutamine, glutamate and aspartate is intensively catabolized by the mucosa of the small intestine. Dietary AA are the main source of energy for the mucosa of the small intestine and precursors for the synthesis of protein, glutathione, polyamines, nitric acids, etc., that play an important role in regulating small intestine functions [10,11].

We hypothesized that dietary Thr and NEAA levels would modify mucin secretion and sugar composition of digesta and crude mucin preparations in different segments of the gut in young pigs. Therefore, the aim of the study was to determine the effect of Thr and NEAA levels on mucin quality and quantity in the gut of young pigs.

## 2. Materials and Methods

### 2.1. Animals and Experimental Procedures

The experimental procedures were approved by the Third Local Animal Experimentation Ethics Committee in Warsaw, Poland (approval numbers 4/2008 and 6/2011). The two-factorial (3 × 4) experiment was conducted on 72 Large White × Duroc male pigs, with an initial body weight (BW) of 12.5 kg. Pigs were allocated to twelve groups of six pigs each.

The animals were housed individually in cages with a slatted floor in a thermally controlled room (22–23 °C) for 20 days and had free access to water. The daily dietary allowance was provided at a rate of about 5% BW. Diets after mixing with water (1:1) were offered in equal portions three times a day at 8:00 a.m., 2:00 p.m., and 8:00 p.m. Pigs were slaughter at a BW of about 22.4 kg.

### 2.2. Diets

The composition and nutritional value of experimental diets were described in detail previously [12]. The diets differed in WG (three levels) and Thr (four levels) content. WG was used as a source of NEAA due to high NEAA content and low Thr content. Three WG levels were obtained by substituting maize starch with 20.4, 40.4 and 60.4 g of WG protein in the WG20, WG40 and WG60 diets, respectively. The latter diets contained an increasing content of total protein: 169, 193 and 213 g/kg, respectively. The diets were supplemented with essential AA (except Thr) to meet pig requirements, as recommended by NRC [13]. Dietary Thr levels were: 5.1, 5.7, 6.3 and 6.9 g of standardized ileal digestible (SID) Thr/kg, which corresponded to 0.51, 0.57, 0.63 and 0.69 of the SID Thr to SID lysine ratio, respectively. According to NRC [13], Thr levels covered 69, 78, 86 and 95% of Thr requirement, respectively, of pigs weighing from 11 to 25 kg BW.

### 2.3. Sampling

At the end of the experiment, the pigs were stunned by an electric shock and exsanguinated. Immediately after slaughter, the abdominal cavity was opened and the entire gastrointestinal tract was removed. The duodenum, middle jejunum, ileum and proximal colon were separated. Intestinal segments were emptied of digesta, rinsed with ice-cold phosphate buffer solution and blotted dry. A mixture of preservatives (15 mM EDTA, 1 mM sodium azide and 2 mM phenylmethylsulfonyl fluoride) was added to fresh digesta of the ileum and proximal colon to reduce enzymatic and bacterial activities. The digesta samples were vigorously shaken, frozen at −20 °C and freeze-dried. Lyophilized digesta was analyzed for the content of crude mucins (luminal crude mucin) and sugars. Digesta for mucin analyses (luminal mucin) were weighted and immediately frozen in liquid nitrogen and stored at −80 °C. The mucosa samples of the duodenum, middle jejunum, ileum and proximal colon were obtained by scraping with a microscope glass slide and immediately freezing in liquid nitrogen and storing at −80 °C for mucin content analyses (tissue mucin).

### 2.4. Crude Mucin Analysis

The content of crude mucins was analyzed only in the digesta of the proximal colon. The amount of digesta collected from the ileum was too low for the determination of luminal crude mucin contents. Therefore, the samples were diluted and luminal crude mucin content was analyzed according to the method described by Piel et al. [14]. Briefly, freeze-dried digesta was suspended in 0.15 M sodium chloride solution and vortexed. The samples were centrifuged at 12,000× *g* for 30 min at 4 °C. The supernatant was mixed with cold ethanol, kept overnight at −20 °C and centrifuged at 1400× *g* for 10 min at 4 °C. The precipitate was dissolved in 0.15 M sodium chloride solution, vortexed and centrifuged again under the same conditions. The precipitate was freeze-dried and weighed.

### 2.5. Mucin Concentration Analysis

#### 2.5.1. Mucin Isolation and Purification

Mucins were isolated and purified using a modified method of Faure et al. [15]. Mucins were extracted from the digesta (luminal mucin) and mucosa (tissue mucin) by reduction and alkylation of disulfide bridges. Briefly, 300 mg of freeze-dried digesta or frozen mucosa were dissolved in 2 mL of ice-cold 5 mM Tris buffer pH 7.5, and homogenized for 45 s at 4 °C at low speed. Next, 0.04 g of Flavourzyme (Novozymes, Bagsværd, Denmark, a fungal complex of exopeptidases and endopeptidases) was added, vortexed for 15 s and incubated with agitation for 18 h at 37 °C. The enzymatic activity of Flavourzyme was stopped by placing the tubes with the homogenate in ice for 10 min. Guanidium hydrochloride and dithiothreitol were subsequently added to a final concentration of 4 M and 10 M, respectively. Then, sample tubes were vortexed and further incubated with agitation for 2 h at room temperature. After adding of iodoacetamide solution (50 mM), the samples were vortexed and incubated with agitation for 2 h at room temperature. Subsequently, the mixtures were centrifuged at 10,000× *g* for 15 min at 4 °C. The supernatant was dialyzed (molecular weight cut-off of dialysis membrane: 12,000–14,000 Da) against deionized water for 48 h at 4 °C. Water was changed twice a day. The obtained solutions were evaporated under nitrogen at 30 °C and stored at −40 °C.

#### 2.5.2. Mucin Assay

Mucin concentration was measured as the content of *O*-linked oligosaccharide chains and assayed according to the method described previously [16]. Briefly, the dried material was dissolved in 120 µL of 10 mM phosphate buffered saline, pH 7.4, and mixed. After adding of 120 µL of alkaline cyanoacetate to 120 µL of the material, the solutions were incubated for 30 min at 100 °C. Then, 1 mL of 0.6 M borate buffer pH 8.0 was added. After cooling, 200 µL of the solutions were transferred to a 96-well black plate with a clear bottom. Fluorescence was measured at 383 nm using a SpectraMax iD3 multi-mode microplate reader (Molecular Devices) at an excitation wavelength of 336 nm.

### 2.6. Sugar Analyses

The content of sugars was determined in the digesta of the ileum and proximal colon and in crude mucin preparations (isolated from the digesta of the proximal colon). Sugar contents were analyzed as their alditol acetate derivatives, according to the procedures described by Lien et al. [17]. Briefly, the digesta samples and crude mucin preparations were hydrolyzed using 12 M sulfuric acid for 1 h at room temperature. The solution was diluted with water to a concentration of 3 M. Hydrolysis was continued for 1 h at 110 °C. Myo-inositol and *N*-methylglucamine internal standards were added to each sample for neutral and acidic sugars, respectively. After hydrolysis, concentrated ammonium hydroxide was added to cold hydrolysates. Next, the samples were reduced for 90 min at 40 °C by adding sodium borohydride solution. The excess sodium borohydride solution was decomposed by adding of concentrated glacial acetic acid, followed by the addition of 1-methylimidiazol and acetic anhydride. The solution was mixed and kept for 10–15 min at room temperature. Subsequently, water was added to degrade the excess acetic anhydride, and the mixture was cooled to room temperature. Alditol acetate derivatives were extracted into dichloromethane by vigorous shaking. The upper aqueous layer was removed. The dichloromethane layer was rinsed with water and evaporated to dryness under a stream of nitrogen. Alditol acetate derivatives were redissolved in dichloromethane. Sugar content was analyzed using a gas chromatograph (GC-2010, Shimadzu, Japan) with a flame-ionization detector (FID) and a DB-17 fused silica column (30 m × 0.25 mm inner diameter × 0.25 µm film thickness). Helium was used as the carrier gas at a flow rate of 1.5 mL/min. Injector temperature was increased from 60 to 270 °C at 150 °C/min and maintained for 20 min. Oven temperature was increased from 50 to 190 °C at 5 °C/min, maintained for 3 min, then elevated to 270 °C at 5 °C/min and maintained for 3 min. FID temperature was set to 270 °C. The samples were analyzed in duplicate. Total run time was approximately 25 min.

### 2.7. Statistical Analyses

Data were statistically evaluated using Statistica 13PL (StatSoft, Kraków, Poland) software. Individual pigs were considered as experimental units. Data were presented as mean ± standard deviation (SD). The model included the effect of the Thr level (four levels: 5.1, 5.7, 6.3 and 6.9 g SID Thr/kg) and WG level (three levels: 20.4, 40.4 and 60.4 g WG protein/kg). The results were statistically evaluated using non-parametric Kruskal–Wallis test. Differences were assessed by non-parametric Mann–Whitney test. Additionally, differences in tissue mucin content along the gut segments (four gut segments: duodenum, middle jejunum, ileum and proximal colon) were analyzed using a non-parametric Kruskal–Wallis test. Differences were evaluated using non-parametric Mann–Whitney test. Differences in the content of mucins and sugars in the digesta of the ileum and proximal colon were analyzed by non-parametric Mann–Whitney test. The level of significance was set at *p* < 0.05 and the trend was defined as 0.05 < *p* < 0.10.

## 3. Results

There was no effect of the Thr level on tissue mucin content in any gut segment (Table 1). The WG level affected tissue mucin content only in the duodenum and middle jejunum. In the duodenum tissue mucin content was higher in the WG60 group compared to the other groups, whereas in the mid-jejunum, it was higher in the WG40 and WG60 groups compared to the WG20 group.

The content of luminal mucins in the ileum and proximal colon was neither affected by Thr nor WG level (Table 2). Dietary WG level influenced luminal crude mucin content in the proximal colon. The content of luminal crude mucins was higher in the WG40 than in the WG60 group.

Unfortunately, fucose, rhamnose, *N*-acetylglucosamine and *N*-acetylgalactosamine were not detected in the digesta samples and crude mucin preparations due to concentration levels below the detection limit of the method used.

Sugar composition of ileal digesta was affected by both Thr and WG levels (Table 3). Ileal digesta of pigs fed diets with the lowest and highest Thr levels contained more glucose and less arabinose and xylose compared to pigs fed diets containing 5.7 and 6.3 g SID Thr per kg. Glucose content in ileal digesta was higher in the WG60 group compared to the WG40 group. The content of arabinose and xylose was higher in the WG20 and WG40 groups in comparison to the WG60 group, while mannose content was higher in the WG20 group than in the WG40 and WG60 groups.

Sugar composition of colonic digesta was not influenced by Thr level (Table 4). WG level only affected the content of arabinose and galactose, which was lower in the WG60 group compared to the WG20 and WG40 groups. Mannose content was not detected in colonic digesta of pigs fed the WG20 and WG40 diets.

Sugar composition of crude mucin preparations isolated from colonic digesta was affected only by Thr level (Table 5). Glucose and galactose contents in crude mucin preparations were lower, whereas arabinose content was higher in pigs fed a diet with the highest Thr level than in pigs fed diets containing 5.7 and 6.3 g SID Thr or all lower Thr contents.

Tissue and luminal mucin contents differed between the gut segments (Table 6). The content of tissue mucin was higher in the ileum and proximal colon and lower in the duodenum than in the middle jejunum. The ileum contained more luminal mucins than the proximal colon. The content of arabinose and glucose was higher, while the content of galactose was lower in the colonic digesta than in the ileal digesta.

## 4. Discussion

### 4.1. Mucin Content in Mucosa and Digesta

Our study found no effect of dietary Thr levels on content of both tissue and luminal mucins in any gut segment of young pigs. These results are consistent with the lack of Thr effect on the number of goblet cells containing all mucin types and the thickness of adherent mucus layer in the small intestine described in the same animals [18]. The negative effect of a prospective high dietary Thr level on the number of acidic goblet cells without changes in the adherent mucus layer thickness was found only in the proximal colon of these pigs [18]. Partially contrasting results were obtained by Law et al. [19], who observed a reduction in tissue crude mucin content in the duodenum and proximal colon, but not in the jejunum and ileum in early-weaned piglets fed a diet with high Thr deficiency (30% of the requirement). Changes in tissue crude mucin content were only partially accompanied by alternations in the number of goblet cell. A reduction in the number of goblet cell containing acidic mucins due to Thr deficiency was observed in the duodenum and ileum, but not in the jejunum and proximal colon [19]. A study of Wellington et al. [20] demonstrated higher total fecal mucin (measured as the amount of *N*-acetylgalactosamine) output in challenged piglets fed a high-Thr, high-fiber diet. However, the number of goblet cell and mucin gene expression were not affected by dietary Thr levels. In addition, Nichols and Bertolo [21] showed that mucin synthesis in the gut of piglets was sensitive to luminal Thr concentration. Generally, the number of goblet cell is recognized as a key parameter describing the ability of the intestinal mucosa to secrete mucin. However, a diet deficient in Thr may directly affect the epithelium and modify mucin release from goblet cells without altering their number.

The results obtained in the present study contradict the well-documented advantageous impact of dietary Thr on the structure and function of pig gut. A large amount of dietary Thr (up to 60%) is utilized by the gut tissue for mucin synthesis [6,7,22]. The results of the present study are also consistent with the lack of low Thr effect on nitrogen retention and gut morphology reported earlier in the same animals [12,23]. This may suggest that a much higher Thr-deficiency than that applied in the present study is necessary to induce changes in mucin secretion. This interpretation is supported by the results of Faure et al. [24], who observed reduced mucin secretion in the ileum only in rats fed a diet with a very low Thr concentration (30% of Thr requirement). On other hand, based on NRC recommendations [13], which were used to formulate the diets, a diet containing 6.3 g Thr/kg was expected to be adequate in terms of Thr level. However, these diets did not cover Thr requirements according to new recommendations [25]. It should also be noted that mucin secretion is influenced not only by low Thr, but also its high levels. However, in the present study, no effect of a prospective high-Thr diet on tissue and luminal mucin contents in the gut was observed. In contrast, a reduction in mucin gene expression in all gut segments of the small intestine, a lower number of goblet cells containing acidic and/or neutral mucins in the duodenum and/or ileum and decreased mucin protein content in the jejunum was observed in weaned pigs fed diets covering both 50 and 150% of the requirement [8,9].

The influence of AA other than Thr on mucin secretion in the pig gut has been studied less frequently. It was shown that diet supplementation with AA other than Thr, but also abundant in mucin proteins, such as serine, cystine and proline, increased the number of goblet cells and tissue mucin content in the ileum and colon in rats with induced colitis [26]. These results have suggested that mucin synthesis may be limited by both low availability of Thr and other AA (serine, cysteine and proline). It should be taken into account that other AA also play important regulatory roles in the gut and are necessary for maintaining optimal gut function. Glutamine and glutamate in particular are involved in many physiological processes, such as the maintenance of intestinal integrity, nutrient metabolism and immune response [27]. Most of dietary glutamine and main part of dietary glutamate and aspartate are utilized by the small intestinal mucosa and used as an energy source by epithelial cells [28].

WG was used as a dietary source of NEAA in the present study due to its high content of these compounds. Glutamic acid is the main NEAA in WG and it can be assumed that it is responsible for the modifying of mucin secretion in the pig gut during WG supplementation.

In the current study, the effect of WG supplementation on tissue and luminal mucin content was found in the duodenum and middle jejunum and in the proximal colon. The duodenum of WG60-fed pigs and the middle jejunum of WG40- and WG60-fed pigs contained more tissue mucin. These results were partially in line with findings of Montagne et al. [29], who demonstrated higher concentration of mucin in duodenal digesta, but not jejunal digesta, of calves fed a diet containing 20.5% crude protein compared to animals fed a diet containing 10.4% crude protein. A further increase of dietary protein content (to 27.8%) only tended to decrease mucin content in duodenal digesta. In the latter study, there was no effect of dietary protein level on mucin content in the digesta in either the jejunum, ileum or colon. However, mucin content in the present study was determined only in the digesta of the ileum and proximal colon. It should be pointed out that the results of our study and the study by Montagne et al. [29] may be difficult to compare as different sample types (mucosa scrapings and digesta), animal species (pigs and calves) and methods (fluorometric assay and ELISA) were applied in both works. Nevertheless, it should be noted that a close correlation between mucin content in rat digesta as measured by fluorometric and ELISA tests was found, which indicated that fluorometric method could be regarded as a useful alternative to ELISA for mucin content determination [30].

Mucin secretion may be also affected by the type of dietary protein. Low-digestible protein products may increase mucin production. It was shown that pea added to the diet stimulated ileal mucin output without changing in the number of goblet cells in the proximal colon of weaned piglets [14,31]. A study of Montagne et al. [29] also demonstrated higher crude mucin flow in the duodenum in calves when skim milk powder was replaced in the diet by plant products (soybean protein concentrate or potato protein concentrate). Moreover, milk protein products were shown to induce mucin release in the jejunum of rats [32,33]. The stimulating effect of protein type could be associated with a higher content of fiber and antinutritional factors in plant feedstuffs and the presence of bioactive peptides in dairy products, which in turn could induce an increase in mucin secretion. However, these explanations were not supported by the results obtained by Lien et al. [34], who failed to find any effect of graded soybean fiber supplementation on mucin production in humans.

The effect of WG supplementation on luminal mucin content in the proximal colon depended on the method of mucin determination, which was absent in case of fluorometry and negative for precipitation. It is difficult to properly interpret our findings. Mucin precipitation using ethanol is considered to be non-specific because the obtained precipitate not only contains raw mucus, but also may be contaminated with other proteins and polysaccharides [35,36]. Fluorometric mucin evaluation is based on the determination of the total amount of *O*-linked oligosaccharide chains [17], which are mucin-specific. This observation may be supported by the findings of Piel et al. [14], who demonstrated large differences in response to the addition of white and black pea to the control diet on mucin output in pigs, as determined by three different methods: ELISA, ethanol precipitation and hexosamine assay. Higher ileal mucin output in piglets fed a diet containing white pea was observed only when the precipitation method was applied. These differences in mucin content in response to dietary factors may suggest that the results of studies on mucin secretion obtained using different analytical methods are not directly comparable.

### 4.2. Sugar Composition in Digesta and Crude Mucin

It is well known that diet composition may affect not only the amount of mucins, but also their composition [37]. Information about sugar composition of mucins may accurately describe the protective functions of the mucus layer.

The content of *N*-acetylgalactosamine and *N*-acetylglucosamine and their ratio are considered useful mucin markers [17,38]. Unfortunately, the content of fucose, rhamnose, *N*-acetylglucosamine and *N*-acetylgalactosamine was not determined in the digesta samples and crude mucin preparations in our study. The content of these sugars in the samples was below the detection limit of the applied method and it was difficult to explain the reason for such a low concentration of these sugars. Fucose and *N*-acetylgalactosamine in ileal mucins in 5-week-old chicken were also not detected; however, the latter study applied a histochemical technique to determine mucin composition [39,40].

In our study, the effect of dietary Thr and WG levels on sugar composition varied depending on the analyzed samples and sugar. Sugar composition in ileal digesta was affected by both Thr and WG levels, and the lowest and highest Thr levels reduced the content of arabinose and xylose, while increased glucose content. The highest WG level reduced the content of arabinose and xylose and increased the content of glucose, while the content of mannose was reduced by both WG40 and WG60 levels. Sugar composition in colonic digesta was affected only by WG levels. WG60 decreased the content of arabinose and galactose. On the other hand, sugar content in colonic crude mucins was affected only by dietary Thr levels. Colonic crude mucins in pigs fed the highest Thr level contained more arabinose, but less glucose and galactose. This indicated that ethanol precipitates contained not only mucins, but also polysaccharides of plant origin. To our knowledge, there are only two published studies concerning the effect of dietary protein on the composition of mucins [41] and digesta [14] in pigs. Sugar content in colonic mucins differed between piglets fed naturally by the mother and piglets fed artificial milk. Colonic mucins of naturally fed animals were found to contain more fucose and glucosamine, but less sulfate and sialic acid [41], which could indicate that colonic mucins of naturally fed piglets provided better protection against intestinal infections. It was reported previously that the addition of white pea, but not black pea to pigs’ diet increased ileal output of glucosamine and galactosamine [14].

### 4.3. Mucin Content and Sugar Composition along the Gut

It is well documented that mucins play a key role in maintaining the protective capacity of the intestinal mucosa. To our knowledge, content of tissue and luminal mucins in individual gut segments of young pigs has not yet been measured. In the present study, tissue mucin content was higher in the ileum and proximal colon and lower in the duodenum than in the middle jejunum. These results are in line with the findings of our previous study [18], which showed a higher number of goblet cells containing all mucin types in the crypts of the ileum and proximal colon in the same piglets. In addition, luminal mucin content was higher in the ileum than in the duodenum and jejunum in calves [37]. Opposite results were obtained by Montagne et al. [42], who observed a similar tissue mucin content in the duodenum, jejunum, ileum and colon in calves. In our study, a higher content of luminal mucins was observed in the ileum than the proximal colon. Mucins are resistant to enzymatic digestion in the small intestine and ileal digesta may contain cumulative mucin content from all segments of the small intestine. It can be assumed that mucins are mainly digested in the colon by bacterial enzymes [38,42]. Differences in sugar composition in the digesta between individual gut segments are difficult to interpret.

## 5. Conclusions

Contrary to expectation, no effect of dietary Thr levels on mucin secretion in the gut of young pigs was found, and it could be assumed that all dietary Thr levels covered the requirements of young pigs for mucin secretion. Wheat gluten added to the diet with adequate Thr content positively affected mucin secretion only in the duodenum and middle jejunum. The effect of wheat gluten on luminal mucin content depended on the analytical method applied. Further research concerning the effect of dietary factors on mucin composition in the pig gut is needed.

## Figures and Tables

**Table 1:** Tissue mucin content measured as the total amount of *O*-linked oligosaccharide chains (µg/g tissue) in gut segments of pigs fed diets differing in standardized ileal digestible threonine (SID Thr) content and wheat gluten (WG) level.

		Gut Segment			
SID Thr, g/kg	WG Level	D	J_50_	I	C_25_
5.1	20	16.47 ± 6.10	24.44 ± 15.00	63.51 ± 27.80	61.56 ± 23.06
	40	15.68 ± 8.53	34.81 ± 10.91	53.52 ± 19.68	61.48 ± 17.21
	60	30.06 ± 13.81	29.03 ± 6.57	57.76 ± 26.08	65.42 ± 34.75
5.7	20	13.01 ± 0.67	36.04 ± 34.99	47.44 ± 19.64	51.14 ± 13.82
	40	17.53 ± 11.21	32.38 ± 23.54	83.31 ± 32.63	58.35 ± 30.07
	60	40.62 ± 28.82	35.43 ± 10.41	69.07 ± 30.22	76.00 ± 30.38
6.3	20	12.41 ± 1.24	24.35 ± 6.29	58.45 ± 16.53	80.87 ± 47.99
	40	13.18 ± 2.11	27.00 ± 8.29	58.38 ± 28.81	56.08 ± 22.31
	60	42.21 ± 18.02	29.46 ± 8.39	66.40 ± 25.37	52.54 ± 24.49
6.9	20	14.01 ± 5.33	22.48 ± 7.04	52.21 ± 21.44	68.11 ± 16.70
	40	27.88 ± 35.74	31.02 ± 12.28	60.13 ± 30.57	61.93 ± 19.40
	60	37.33 ± 32.69	42.19 ± 29.72	79.40 ± 24.48	59.64 ± 21.47
Mean		23.52	30.70	62.37	62.83
Pooled SEM		5.28	5.46	9.64	9.58
SID Thr, g/kg					
5.1		20.33 ± 11.42	29.43 ± 11.69	58.26 ± 24.03	62.82 ± 24.96
5.7		24.32 ± 21.92	34.66 ± 23.08	66.73 ± 30.41	62.57 ± 27.05
6.3		23.63 ± 17.96	27.07 ± 7.63	61.35 ± 23.17	62.61 ± 33.80
6.9		26.33 ± 28.00	31.94 ± 20.15	64.10 ± 27.00	63.29 ± 18.62
WG level					
20		13.96 ± 4.50 ^a^	26.49 ± 18.51 ^a^	55.82 ± 21.92	65.23 ± 28.21
40		18.35 ± 18.24 ^a^	31.57 ± 14.03 ^b^	63.04 ± 28.44	59.62 ± 21.08
60		37.56 ± 23.65 ^b^	33.85 ± 16.37 ^b^	67.80 ± 26.37	63.47 ± 28.30
*p*-value					
SID Thr		0.987	0.867	0.448	0.432
WG level		0.001	0.002	0.250	0.618

Gut segments: D—duodenum, J_50_—middle jejunum, I—ileum, C_25_—proximal colon; SID Thr—standardized ileal digestible threonine; WG—wheat gluten; SEM—standard error of the mean; results are presented as mean values ± standard deviation (SD); ^a,b^—means with different superscripts within a column are significantly different at *p* ≤ 0.05.

**Table 2:** Luminal mucin content measured as total amount of *O*-linked oligosaccharide chains (µg/g digesta) and luminal crude mucin content (%) in the ileum and proximal colon of pigs fed diets differing in standardized ileal digestible threonine (SID Thr) content and wheat gluten (WG) level.

		Mucin (*O*-Linked Oligosaccharide Chains)		Crude Mucin
SID Thr, g/kg	WG level	I	C_25_	C_25_
5.1	20	457.6 ± 235.5	304.0 ± 105.0	5.36 ± 1.45
	40	617.8 ± 298.8	345.8 ± 65.7	6.30 ± 1.62
	60	441.2 ± 178.3	338.1 ± 120.1	4.62 ± 1.20
5.7	20	654.5 ± 406.6	281.8 ± 57.68	5.31 ± 1.36
	40	571.7 ± 300.8	312.9 ± 45.6	6.09 ± 1.29
	60	630.0 ± 369.3	344.1 ± 85.5	4.53 ± 1.36
6.3	20	597.7 ± 289.7	312.1 ± 52.7	4.86 ± 1.19
	40	476.5 ± 191.6	313.3 ± 48.0	5.34 ± 1.10
	60	399.8 ± 172.4	324.5 ± 81.9	4.59 ± 1.46
6.9	20	476.7 ± 138.1	287.7 ± 59.9	5.80 ± 2.86
	40	683.5 ± 295.7	353.2 ± 60.0	5.88 ± 1.20
	60	472.7 ± 130.6	398.1 ± 83.7	5.06 ± 1.06
Mean		534.8	327.3	5.30
Pooled SEM		97.2	28.2	0.55
SID Thr, g/kg				
5.1		505.5 ± 245.7	329.3 ± 97.0	5.43 ± 1.55
5.7		619.3 ± 343.1	314.6 ± 69.2	4.00 ± 1.43
6.3		486.5 ± 224.7	317.0 ± 60.6	3.73 ± 1.24
6.9		535.9 ± 206.6	346.0 ± 81.0	4.45 ± 1.86
WG level				
20		542.3 ± 280.1	296.63 ± 71.5	5.35 ± 1.79 ^a,b^
40		586.0 ± 269.2	333.2 ± 56.3	5.93 ± 1.31 ^b^
60		484.4 ± 234.2	350.8 ± 94.3	4.70 ± 1.24 ^a^
*p*-value				
SID Thr		0.475	0.145	0.991
WG level		0.205	0.242	0.017

Gut segments: I—ileum, C_25_—proximal colon; SID Thr—standardized ileal digestible threonine; WG—wheat gluten; SEM—standard error of the mean; results are presented as mean values ± standard deviation (SD); ^a,b^—means with different superscripts within a column are significantly different at *p* ≤ 0.05.

**Table 3:** Sugar composition (mol/100 mol) in ileal digesta of pigs fed diets differing in standardized ileal digestible threonine (SID Thr) content and wheat gluten (WG) level.

SID Thr, g/kg	WG Level	Arabinose	Xylose	Mannose	Glucose	Galactose
5.1	20	20.31 ± 3.15	29.83 ± 4.46	3.51 ± 1.29	41.16 ± 10.72	6.95 ± 3.29
	40	20.04 ± 1.48	29.01 ± 2.09	2.08 ± 1.07	42.37 ± 5.67	7.30 ± 2.95
	60	19.14 ± 0.99	27.04 ± 1.89	n.d.	47.39 ± 3.13	6.20 ± 1.39
5.7	20	21.94 ± 0.57	34.23 ± 1.98	2.75 ± 1.47	36.66 ± 2.22	5.76 ± 1.34
	40	20.69 ± 1.57	30.41 ± 2.67	1.49 ± 0.49	42.35 ± 5.00	6.06 ± 1.14
	60	20.14 ± 0.70	28.66 ± 1.73	2.10 ± 1.53	45.44 ± 1.84	5.66 ± 0.89
6.3	20	21.34 ± 0.86	31.07 ± 2.14	2.52 ± 1.39	38.30 ± 2.75	7.33 ± 2.19
	40	21.30 ± 1.71	30.82 ± 2.17	n.d.	41.07 ± 4.18	6.67 ± 1.73
	60	20.05 ± 1.80	29.26 ± 3.47	0.90 ± 0.26	44.72 ± 4.77	5.83 ± 0.92
6.9	20	20.39 ± 2.11	28.75 ± 3.20	2.77 ± 1.32	42.48 ± 4.76	6.69 ± 1.28
	40	19.40 ± 2.16	29.01 ± 2.01	0.49 ± 0.07	46.07 ± 4.86	5.62 ± 1.42
	60	18.49 ± 1.80	25.87 ± 3.47	0.82 ± 0.26	50.34 ± 4.77	5.11 ± 0.92
Mean		20.21	29.36	2.06	43.35	6.29
Pooled SEM		0.60	0.94	0.44	1.74	0.65
SID Thr, g/kg						
5.1		19.83 ± 2.06 ^a^	28.63 ± 3.15 ^a^	2.58 ± 1.27	43.64 ± 7.44 ^b^	6.82 ± 2.60
5.7		20.89 ± 1.24 ^b^	30.97 ± 3.13 ^b^	2.17 ± 1.29	41.69 ± 4.85 ^a^	5.82 ± 1.07
6.3		20.79 ± 1.52 ^b^	30.24 ± 2.02 ^b^	1.96 ± 1.29	41.62 ± 4.41 ^a^	6.57 ± 1.76
6.9		19.38 ± 2.06 ^a^	27.70 ± 3.24 ^a^	1.58 ± 1.34	46.53 ± 5.66 ^b^	5.78 ± 1.32
WG level						
20		20.94 ± 2.09 ^b^	30.88 ± 3.67 ^b^	2.85 ± 1.30 ^b^	39.70 ± 6.66 ^a,b^	6.70 ± 2.24
40		20.37 ± 1.74 ^b^	29.78 ± 2.26 ^b^	1.63 ± 0.96 ^a^	42.80 ± 5.03 ^a^	6.51 ± 2.05
60		19.41 ± 1.41 ^a^	27.63 ± 2.47 ^a^	1.31 ± 0.99 ^a^	47.04 ± 3.93 ^b^	5.72 ± 1.16
*p*-value						
SID Thr		0.006	0.008	0.109	0.015	0.382
WG level		0.003	0.001	0.001	0.001	0.190

SID Thr—standardized ileal digestible threonine; WG—wheat gluten; SEM—standard error of the mean; results are presented as mean values ± standard deviation (SD); ^a,b^—means with different superscripts within a column are significantly different at *p* ≤ 0.05; n.d.—not determined.

**Table 4:** Sugar composition (mol/100 mol) in colonic digesta of pigs fed diets differing in standardized ileal digestible threonine (SID Thr) content and wheat gluten (WG) level.

SID Thr, g/kg	WG Level	Arabinose	Xylose	Mannose	Glucose	Galactose
5.1	20	20.85 ± 1.29	29.82 ± 2.77	n.d.	47.34 ± 2.68	6.17 ± 3.42
	40	22.70 ± 3.22	33.01 ± 5.22	n.d.	46.77 ± 5.77	4.47 ± 0.96
	60	19.77 ± 1.34	28.73 ± 2.20	2.50 ± 0.34	48.45 ± 2.93	3.14 ± 0.43
5.7	20	22.09 ± 1.32	32.06 ± 2.48	n.d.	45.85 ± 3.23	n.d.
	40	21.74 ± 1.52	30.17 ± 2.91	n.d.	44.84 ± 2.19	5.04 ± 1.01
	60	19.96 ± 2.36	29.01 ± 2.98	n.d.	50.48 ± 5.02	n.d.
6.3	20	21.00 ± 1.88	29.95 ± 1.76	n.d.	47.93 ± 3.42	4.46 ± 0.47
	40	22.89 ± 5.16	31.89 ± 7.94	n.d.	49.90 ± 5.89	5.32 ± 1.18
	60	20.22 ± 1.55	28.93 ± 1.51	2.67 ± 0.10	48.92 ± 3.67	4.84 ± 0.54
6.9	20	20.89 ± 1.58	30.78 ± 3.23	n.d.	47.07 ± 4.23	4.93 ± 0.83
	40	20.85 ± 1.34	30.16 ± 3.34	n.d.	46.96 ± 2.66	4.32 ± 0.21
	60	19.60 ± 0.87	29.71 ± 1.91	n.d.	48.99 ± 1.91	4.17 ± 0.54
Mean		21.02	30.36	2.58	47.72	4.54
Pooled SEM		0.74	1.21	0.10	1.39	0.46
SID Thr, g/kg						
5.1		21.11 ± 2.39	30.52 ± 3.94	2.50 ± 0.34	47.52 ± 3.93	4.32 ± 2.05
5.7		21.19 ± 1.98	30.34 ± 2.95	n.d.	47.24 ± 4.39	4.60 ± 1.41
6.3		21.49 ± 3.18	30.35 ± 4.51	2.67 ± 0.10	48.47 ± 4.28	4.96 ± 0.97
6.9		20.42 ± 1.37	30.22 ± 2.76	n.d.	47.71 ± 3.10	4.42 ± 0.57
WG level						
20		21.17 ± 1.51 ^b^	30.60 ± 2.65	n.d.	47.07 ± 3.29	5.29 ± 2.17 ^b^
40		22.09 ± 3.12 ^b^	31.44 ± 5.08	n.d.	47.09 ± 4.67	4.79 ± 0.94 ^b^
60		20.00 ± 1.58 ^a^	29.19 ± 2.13	2.58 ± 0.31	48.89 ± 3.50	3.71 ± 0.95 ^a^
*p*-value						
SID Thr		0.387	0.668	0.311	0.661	0.195
WG level		0.004	0.107	-	0.085	0.004

SID Thr—standardized ileal digestible threonine; WG—wheat gluten; SEM—standard error of the mean; results are presented as mean values ± standard deviation (SD); ^a,b^—means with different superscripts within a column are significantly different at *p* ≤ 0.05; n.d.—not determined.

**Table 5:** Sugar composition (mol/100 mol) of crude mucins preparations isolated from colonic digesta of pigs fed diets differing in standardized ileal digestible threonine (SID Thr) content and wheat gluten (WG) level.

SID Thr, g/kg	WG Level	Arabinose	Xylose	Mannose	Glucose	Galactose
5.1	20	20.54 ± 4.80	17.69 ± 5.96	5.79 ± 5.10	44.51 ± 5.16	24.93 ± 5.86
	40	22.43 ± 5.64	22.31 ± 3.27	n.d.	41.14 ± 9.57	24.63 ± 6.75
	60	18.46 ± 3.36	15.55 ± 5.39	5.69 ± 4.22	51.73 ± 10.27	23.60 ± 5.82
5.7	20	20.02 ± 4.44	15.82 ± 6.88	11.96 ± 8.54	45.05 ± 4.09	26.78 ± 7.78
	40	19.04 ± 1.64	16.82 ± 3.07	14.19 ± 8.43	43.20 ± 6.76	22.74 ± 5.37
	60	16.22 ± 5.25	19.29 ± 7.40	10.82 ± 2.88	47.38 ± 6.59	19.90 ± 7.82
6.3	20	17.66 ± 3.72	16.87 ± 5.15	n.d.	50.40 ± 11.68	23.23 ± 3.76
	40	16.75 ± 2.15	16.85 ± 4.85	4.57 ± 3.28	45.04 ± 4.90	23.94 ± 2.80
	60	17.96 ± 3.39	16.71 ± 6.15	n.d.	47.56 ± 5.69	21.73 ± 4.35
6.9	20	21.58 ± 7.34	19.25 ± 4.24	n.d.	39.67 ± 12.47	16.86 ± 4.31
	40	19.92 ± 1.85	20.61 ± 3.21	n.d.	40.98 ± 4.68	18.32 ± 4.56
	60	22.34 ± 3.13	27.73 ± 6.17	9.39 ± 4.57	39.14 ± 6.84	18.17 ± 7.26
Mean		19.46	18.71	9.60	44.76	22.13
Pooled SEM		1.58	2.28	2.49	2.98	2.24
SID Thr, g/kg						
5.1		20.38 ± 4.68 ^a,b^	17.77 ± 5.55	5.75 ± 3.74	46.03 ± 9.28 ^a,b^	24.37 ± 5.82 ^b^
5.7		18.21 ± 4.24 ^a^	17.50 ± 5.75	12.25 ± 5.87	45.34 ± 6.00 ^b^	22.76 ± 7.24 ^b^
6.3		17.77 ± 3.10 ^a^	17.09 ± 5.02	8.11 ± 7.56	47.89 ± 7.86 ^b^	23.13 ± 3.54 ^b^
6.9		21.28 ± 4.56 ^b^	22.62 ± 5.72	9.80 ± 7.41	39.93 ±8.16 ^a^	17.76 ± 7.26 ^a^
WG level						
20		19.97 ± 5.13	17.39 ± 5.26	9.93 ± 7.13	44.89 ± 9.40	22.87 ± 6.38
40		19.53 ± 3.68	18.70 ± 4.13	8.62 ± 7.66	42.59 ± 6.53	22.41 ± 5.36
60		18.90 ± 4.33	19.81 ± 7.47	10.30 ± 4.57	46.64 ± 8.52	21.15 ± 6.39
*p*-value						
SID Thr		0.039	0.061	0.189	0.044	0.004
WG level		0.907	0.561	-	0.136	0.720

SID Thr—standardized ileal digestible threonine; WG—wheat gluten; SEM—standard error of the mean; results are presented as mean values ± standard deviation (SD); ^a,b^—means with different superscripts within a column are significantly different at *p* ≤ 0.05; n.d.—not determined.

**Table 6:** Tissue and luminal mucin content (µg/g tissue or digesta) and digesta sugar composition (mol/100 mol) in gut segments of young pigs.

Item	Gut Segment				Pooled	*p*-Value
	D	J_50_	I	C_25_	SEM	
Tissue mucin, µg/g tissue	23.52 ± 15.53 ^a^	30.70 ± 16.33 ^b^	62.37 ±25.56 ^c^	62.83 ± 25.98 ^c^	2.49	0.001
Luminal mucin, µg/g digesta	-	-	534.8 ± 259.8 ^b^	327.3 ± 75.0 ^a^	21.42	0.001
Sugar composition of digesta, mol/100 mol						
arabinose	-	-	20.21 ± 1.85 ^a^	21.05 ± 2.31 ^b^	0.23	0.013
xylose	-	-	29.36 ± 3.15	30.36 ± 3.56	0.37	0.105
mannose	-	-	2.06 ± 1.31	2.58 ± 0.31	0.38	0.104
glucose	-	-	43.35 ± 6.04 ^a^	47.72 ± 3.90 ^b^	0.58	0.001
galactose	-	-	6.29 ± 1.88 ^b^	4.54 ± 1.42 ^a^	0.23	0.001

Gut segments: D—duodenum, J_50_—middle jejunum, I—ileum, C_25_—proximal colon; SEM—standard error of the mean; results are presented as mean values ± standard deviation (SD); ^a,b,c^—means with different superscripts within a row are significantly different at *p* ≤ 0.05.

## Data Availability

The data presented in the study are available on request from the corresponding author.
